# Innovation at the Edge of Nutrition Education Research

**DOI:** 10.3390/nu13062018

**Published:** 2021-06-11

**Authors:** Lauren Ball, Amy Kirkegaard

**Affiliations:** 1 Menzies Health Institute of Queensland, Griffith University, Southport, QLD 4111, Australia; a.kirkegaard@griffith.edu.au; 2 Nutrition and Dietetics, Gold Coast Campus, School of Health Sciences and Social Work, Griffith University, Southport, QLD 4111, Australia

The fundamental role of diet in maximizing human health and utility is now universally recognized [1]. In turn, governments and healthcare services around the world are acting to maximize their ability to improve the health of the people in their care through food and nutrition initiatives [2]. Hospitals now routinely enlist the support of community volunteers to help with food preparation and tailored feeding of patients [3]. In the community, practice guidelines for health professionals position dietary changes as the first line treatment for chronic conditions such as type 2 diabetes and cardiovascular disease [4]. Doctors, nurses, and other health professionals are increasingly undertaking nutrition education courses to better equip themselves to create positive health outcomes for patients [5,6]. The UN Decade of Action on Nutrition (2016–2025) is now at the half-way mark, and a review of progress is underway via their Foresight paper [7], with major achievements likely to be recognized across food systems, health systems, social protection, and trade and environments that support healthy eating.

Research on nutrition in health services and nutrition workforces is maturing within our increasingly VUCA world; volatile, uncertain, complex, and ambiguous [8]. We are now seeing research appropriately sit at the edge of traditional boundaries, working on the discipline’s evolution so that it grows the positive impact that nutrition has on society. The papers in this special issue are another example of such research reflecting transformation, because they are collectively furthering nutrition care and innovation, pushing boundaries so that more people can access support to eat well. Congratulations to these authors on their anticipation, insight, and preparedness, paving the way for future trends in applied nutrition research.

Three of the papers in this Special Issue are capitalizing on the easy and free access to nutrition information that has never been more accessible to the general public. With the increasingly prominent role that the internet plays in our lives, more and more people are turning to online sources for information on food, diet, and nutrition. Unfortunately, this largely unmoderated environment is also home to information that is not evidence-based. Discerning the quality of nutrition information sources can pose a challenge for the general public. Massive Open Online Courses (MOOCs), such as the ‘Food as Medicine’ course described in three papers in this special issue, are a promising and increasingly popular way to address this issue. The study by Gibson and colleagues highlights the attractiveness of this educational mode to the public and contributes important lessons for individuals seeking to develop MOOCs targeting the general public in the future [9]. Adamski and colleagues contribute further evidence relating to consumers of nutrition-related MOOCs and their information-seeking behaviors, which is pertinent to professionals looking to engage with the public via this mode [10]. Similarly, Cowan and colleagues focused on learners’ perspectives of nutrition and inflammation and identify opportunities for health professionals to employ innovative education strategies when engaging with the public [11]. Collectively, these studies highlight the value and relevance of MOOCs in nutrition education and the opportunity to meet the public’s thirst for evidence-based nutrition information.

From education of the public to education of patients in the healthcare setting, countless studies have described patients’ desire for nutrition care from health professionals. We know that healthcare consumers’ demand for consistent, evidence-based, up-to-date nutrition advice from their healthcare team is ever expanding [12]. In this Special Issue, the study by Lepre and colleagues explores medical students’ readiness to meet this demand, and reports that the soon-to-be members of the medical profession value the roles of both doctors and the multidisciplinary team in providing nutrition advice [13]. Similarly, the study by Zhang and colleagues explored dietary advice of optometrists in Australia and New Zealand and, encouragingly, found that this practice is often routine [14]. However, both studies identified that knowledge and skill deficiencies were reported by participants, suggesting that innovative ways to support these professionals to translate research into the clinical setting are needed. Dietitians play an important role in the provision of nutrition advice in primary care and were the focus of the study by Porter and colleagues [15]. Innovative approaches to nutrition and dietetics education that incorporated patients were explored in this review, with evidence in support of enhanced student learning reported. Innovation in health professional education, both in the university and professional learning settings, play a key role in supporting healthcare professionals to provide nutrition advice to meet patients’ needs.

This Special Issue has sparked several opportunities for future research and we look forward to seeing more cutting-edge work from emerging and advancing academics and practitioners.

## Data Availability

Not applicable.

## References

[1] United Nations Decade of Action on Nutrition Secretariat United Nations Decade of Action on Nutrition: 2016–2025. https://www.who.int/nutrition/decade-of-action/info_flyer.pdf?ua=1.

[2] Downer S., Berkowitz S.A., Harlan T.S., Olstad D.L., Mozaffarian D. (2020). Food is medicine: Actions to integrate food and nutrition into healthcare. BMJ.

[3] Howson F.F.A., Robinson S.M., Lin S.X., Orlando R., Cooper C., Sayer A.A.P., Roberts H.C. (2018). Can trained volunteers improve the mealtime care of older hospital patients? An implementation study in one English hospital. BMJ Open.

[4] Ball L., Leveritt M., Cass S., Chaboyer W. (2015). Effect of nutrition care provided by primary health professionals on adults’ dietary behaviours: A systematic review. Fam. Pract..

[5] Crowley J., Ball L., Hiddink G.J. (2019). Nutrition in medical education: A systematic review. Lancet Planet Health.

[6] Cass S., Ball L., Leveritt M. (2014). Australian practice nurses’ perceptions of their role and competency to provide nutrition care to patients living with chronic disease. Aust. J. Prim. Health.

[7] United Nations System Standing Committee on Nutrition Mid-Term Review of the UN Decade of Action on Nutrition—Foresight Paper. https://www.unscn.org/en/topics/un-decade-of-action-on-nutrition?idnews=2038.

[8] Academy of Nutrition and Dietetics Navigating Future Practice: VUCA. https://www.eatrightidaho.org/app/uploads/2021/02/xta/0/VUCA-change-driver.pdf.

[9] Gibson S., Adamski M., Blumfield M., Dart J., Murgia C., Volders E., Truby H. (2020). Promoting Evidence Based Nutrition Education Across the World in a Competitive Space: Delivering a Massive Open Online Course. Nutrients.

[10] Adamski M., Truby H., Klassen K.M., Cowan S., Gibson S. (2020). Using the Internet: Nutrition Information-Seeking Behaviours of Lay People Enrolled in a Massive Online Nutrition Course. Nutrients.

[11] Cowan S., Sood S., Truby H., Dordevic A., Adamski M., Gibson S. (2020). Inflaming Public Interest: A Qualitative Study of Adult Learners’ Perceptions on Nutrition and Inflammation. Nutrients.

[12] Ball L., Desbrow B., Leveritt M. (2014). An exploration of individuals’ preferences for nutrition care from Australian primary care health professionals. Aust. J. Prim. Health.

[13] Lepre B., Crowley J., Mpe D., Bhoopatkar H., Mansfield K.J., Wall C., Beck E.J. (2020). Australian and New Zealand Medical Students’ Attitudes and Confidence Towards Providing Nutrition Care in Practice. Nutrients.

[14] Zhang A.C., Singh S., Craig J.P., Downie L.E. (2020). Omega-3 Fatty Acids and Eye Health: Opinions and Self-Reported Practice Behaviors of Optometrists in Australia and New Zealand. Nutrients.

[15] Porter J., Kellow N., Anderson A., Bryce A., Dart J., Palermo C., Volders E., Gibson S. (2019). Patient Involvement in Education of Nutrition and Dietetics Students: A Systematic Review. Nutrients.

